# Stopping targeted therapy for complete responders in advanced BRAF mutant melanoma

**DOI:** 10.1038/s41598-020-75837-5

**Published:** 2020-11-02

**Authors:** L. Warburton, T. M. Meniawy, L. Calapre, M. Pereira, A. McEvoy, M. Ziman, E. S. Gray, M. Millward

**Affiliations:** 1 Department of Medical Oncology, Sir Charles Gairdner Hospital, Nedlands, WA Australia; 2 School of Medical and Health Sciences, Edith Cowan University, Joondalup, WA Australia; 3 School of Medicine and Pharmacology, University of Western Australia, Crawley, WA Australia; 4 School of Biomedical Science, University of Western Australia, Crawley, WA Australia

**Keywords:** Cancer therapy, Targeted therapies, Oncology

## Abstract

BRAF inhibitors revolutionised the management of melanoma patients and although resistance occurs, there is a subgroup of patients who maintain durable disease control. For those cases with durable complete response (CR) it is not clear whether it is safe to cease therapy. Here we identified 13 patients treated with BRAF +/− MEK inhibitors, who cease therapy after prolonged CR (median = 34 months, range 20–74). Recurrence was observed in 3/13 (23%) patients. In the remaining 10 patients with sustained CR off therapy, the median follow up after discontinuation was 19 months (range 8–36). We retrospectively measured ctDNA levels using droplet digital PCR (ddPCR) in longitudinal plasma samples. CtDNA levels were undetectable in 11/13 cases after cessation and remained undetectable in patients in CR (10/13). CtDNA eventually became detectable in 2/3 cases with disease recurrence, but remained undetectable in 1 patient with brain only progression. Our study suggests that consideration could be given to ceasing targeted therapy in the context of prolonged treatment, durable response and no evidence of residual disease as measured by ctDNA.

## Introduction

The identification of oncogenic mutations in BRAF led to the development of therapeutic options for patients with advanced melanoma^1^. BRAF activating mutations, found in 40–50% of melanomas, result in activation of the Mitogen-activated protein kinases (MAPK) pathway. Highly specific mutant BRAF inhibitors have been approved for clinical use—vemurafenib, encorafenib and dabrafenib^2,3^. These drugs have improved response rates, progression free survival (PFS) and overall survival (OS) in patients with stage IV BRAF mutant metastatic melanoma^4^. The combination of BRAF inhibitors with MEK inhibitors has translated to the delayed evolution of resistance, resulting in improved progression free survival and outcomes for these patients^5–9^. Four and five year OS has been shown to be around 30–40%, with PFS approximating 15%, demonstrating durable survival which appears to plateau at 4 years^10,11^. Patients with good prognostic features such as normal lactate dehydrogenase levels, low volume disease and good performance status had higher rates of durable control and survival^11–13^. In the 5 year landmark analysis of COMBI-d, the patients who remained progression free at time of analysis, 88% remained on combination BRAF and MEK inhibition^11^. Targeted therapies for melanoma are relatively well tolerated with an excellent safety profile. It is unclear whether stopping targeted therapy in the setting of a complete response (CR) is a safe option. However, as healthcare costs continue to rise there is an increasing interest in the assessment of the cost–benefit of current regimes. Given the significant financial burden of these treatments, identifying patients that may be able to stop therapy in the absence of disease progression or toxicity should be evaluated.

Circulating tumour-derived cell free DNA (ctDNA) has been investigated as a potential biomarker of disease status in advanced melanoma patients. Short nucleic fragments, carrying genetic information including BRAF V600 mutations, are released by tumours allowing for blood based monitoring^14^. Studies in colorectal cancer have shown the utility of ctDNA in measuring minimal residual disease as a predictor of recurrence and a way to stratify patients requiring additional therapy^15^. Recent reports showed that ctDNA detection in plasma taken within 12 weeks of curative intent surgery is highly predictive of relapse in patients with stage II and III melanoma^16,17^. CtDNA responses in melanoma patients who have achieved a complete radiological response may be able to aid clinical decision making to determine if cessation of treatment is a viable option.

We present the outcomes of a case series of 13 patients who achieved complete tumour response following BRAF inhibitor monotherapy or combination BRAF/MEK inhibition, and ceased therapy for observation after maintaining sustained remission. This is a unique cohort as we have not included patients whom ceased due to toxicity. In addition, we analysed plasma samples from thirteen of these patients for the presence of BRAF mutant ctDNA as an indicator of disease presence.

## Material and methods

### Study design, setting and participants

We retrospectively reviewed the medical records of all patients with BRAF mutant metastatic melanoma who received first line BRAF inhibition monotherapy (vemurafenib, encorafenib or dabrafenib) or combination BRAF/MEK therapy (dabrafenib and trametinib) treated at Sir Charles Gairdner Hospital, Perth, Western Australia from November 2009 to January 2019. We included only those patients that discontinued treatment after achieving a CR and had a minimum time on therapy of 18 months. We excluded patients who stopped due to progressive disease or toxicity. The treatment was stopped after extensive discussion with the treating oncologist about the risks and benefits or at the request of the patient. The following information was collected: age, gender, Eastern Cooperative Oncology group (ECOG) performance status, melanoma stage, number of metastatic sites, LDH at baseline, therapy instituted, duration of treatment, time to CR, days off therapy and any toxicity reported. Recurrence was defined by disease recurrence at any site during observation according to standard RECIST criteria.

### Blood collection

Plasma samples were collected from 13 patients analysed at various time points either during and/or prior to and beyond cessation of therapy or beyond cessation of therapy only. Samples were provided between April 2013 and May 2018. Written informed consent was obtained from all patients. The study was conducted according to the principles set out by the National Statement on Ethical Conduct in Human Research and Good Clinical Practice Guidelines by the National Health and Medical Research Council.

Blood collection and ctDNA analysis were perfomed as described previously in other publications from our laboratory^18–20^. Patient peripheral blood samples were collected in EDTA and Streck tubes. Plasma was separated within 24 h by centrifugation at 300×*g* for 20 min, followed by a second centrifugation at 4700×*g* for 10 min, and then stored at – 80 °C until extraction. The cell free DNA (cfDNA) was extracted from 1 to 5 mL of plasma using the QIAamp Circulating Nucleic Acid Kit (Qiagen) as per the manufacturer’s instructions. Extracted samples were then frozen until analysis. The ctDNA was quantified by droplet digital PCR as previously described^18,19^. Amplifications were carried out in a 20 μL reaction containing droplet PCR supermix, primers, probe and cfDNA. Samples were analysed for BRAF V600E or V600K mutations depending on the mutation identified in the patient’s biopsy. Droplets were generated and analysed using the QX200 system (Bio-Rad). Samples were analysed in triplicate, and considered positive if at least one triplicate was positive.

### Ethical approval and consent to participate

The study was approved by the Human Research Ethics Committee of Edith Cowan University (No. 2932) and Sir Charles Gairdner Hospital (No. 2007-123).

## Results

### Patient characteristics and response to treatment

A total of thirteen patients that met the inclusion criteria were identified (Table 1). The median age was 61 years (38–71) and 54% were males. The baseline ECOG performance status was 0 in 11 patients. Two patients had baseline LDH greater than the upper limit of normal. There were three patients who had Stage M1a metastatic disease, one with M1b disease, five with M1c and four with M1d disease as per the AJCC TNM cancer staging system (8th edition). Three patients had more than three metastatic sites of disease. Four patients had brain metastasis at baseline; three of these patients had surgical excision with no radiological residual intracranial disease evident at commencement of therapy.

**Table 1:** Patient cohort characteristics and outcome of patients treated with BRAF inhibition.

Pt no.	Sex	Age	Stage AJCC 8th	Mutation	LDH	Met sites	Location	Therapy	Dose reduction	Toxicity	PD?	Time to relapse after cessation (months)	Site of PD	Re- treatment	Response to re-challenge
1	F	42	M1c	V600K	< ULN	< 3	Lung, bones	Dabrafenib	–	–	No	–	–	–	–
2	M	70	M1c	V600K	> 2 × ULN	> 3	Liver, lung, bones	CombiDT	–	–	No	–	–	–	–
3	F	68	M1c	V600E	< ULN	> 3	Nodal, adrenal, intra-abdominal	Dabrafenib	–	–	No	–	–	–	–
4	M	62	M1c	V600E	< ULN	< 3	Liver, spleen	CombiDT	–	–	No	–	–	–	–
5	M	60	M1d	V600E	< ULN	< 3	Brain (resected), lung	Vemurafenib	–	–	No	–	–	–	–
6	**M**	**67**	**M1c**	**V600E**	** < ULN**	** < 3**	**Nodal, soft tissue, bone**	**Encorafenib**	**Yes**	**Skin Toxicity**	**Yes**	**5**	**Brain**	**CombiDT**	**CR**
7	**F**	**45**	**M1d**	**V600E**	** < ULN**	** > 3**	**Brain, liver, nodal**	**CombiDT**	**Yes**	Deranged LFTs and Flare of IBD*	**Yes**	**5**	**Brain**	**CombiDT**	**PR**
8	**M**	**61**	**M1d**	**V600E**	** < ULN**	** < 3**	**Brain (resected), nodal**	**CombiDT**	–	–	**Yes**	**11**	**Nodal**	**CombiDT**	**CR**
9	M	66	M1b	V600E	< ULN	< 3	Lung	CombiDT	–	–	No	–	–	–	–
10	F	37	M1a	V600E	< ULN	< 3	Nodal, subcutaneous	Vemurafenib	–	–	No	–	–	–	–
11	F	42	M1d	V600E	> ULN	< 3	Brain, nodal	Dabrafenib	–	–	No	–	–	–	–
12	F	55	M1a	V600E	< ULN	< 3	Nodal	Dabrafenib	–	–	No	–	–	–	–
13	M	43	M1a	V600E	< ULN	> 3	Nodal, subcutaneous	CombiDT	–	–	No	–	–	–	–

*AJCC* American joint committee on cancer 8th edition, *PD* Progressive disease.

*Patient 7 had a pre-existing diagnosis of ulcerative colitis which had remained quiescent prior to targeted therapy. Commencement of full dose combiDT flared diarrhoea and settled with dose reduction.

Bold rows indicate patients that progressed after cessation of therapy.

The patients all had confirmed *BRAF* V600E/K mutation in their melanoma on molecular analysis. Two patients had a V600K mutation and the rest were V600E mutant as tested by Sanger Sequencing on the original metastatic confirmatory biopsy. BRAF inhibition was the first line therapy in all 13 patients, with six patients treated with combination dabrafenib and trametinib, one patient received encorafenib, four received dabrafenib monotherapy and two received vemurafenib alone. Two patients required dose reductions for toxicity. They all achieved a CR to therapy. The mean time to CR was 9 months (median: 8, range 1–23).

**Figure 1:**
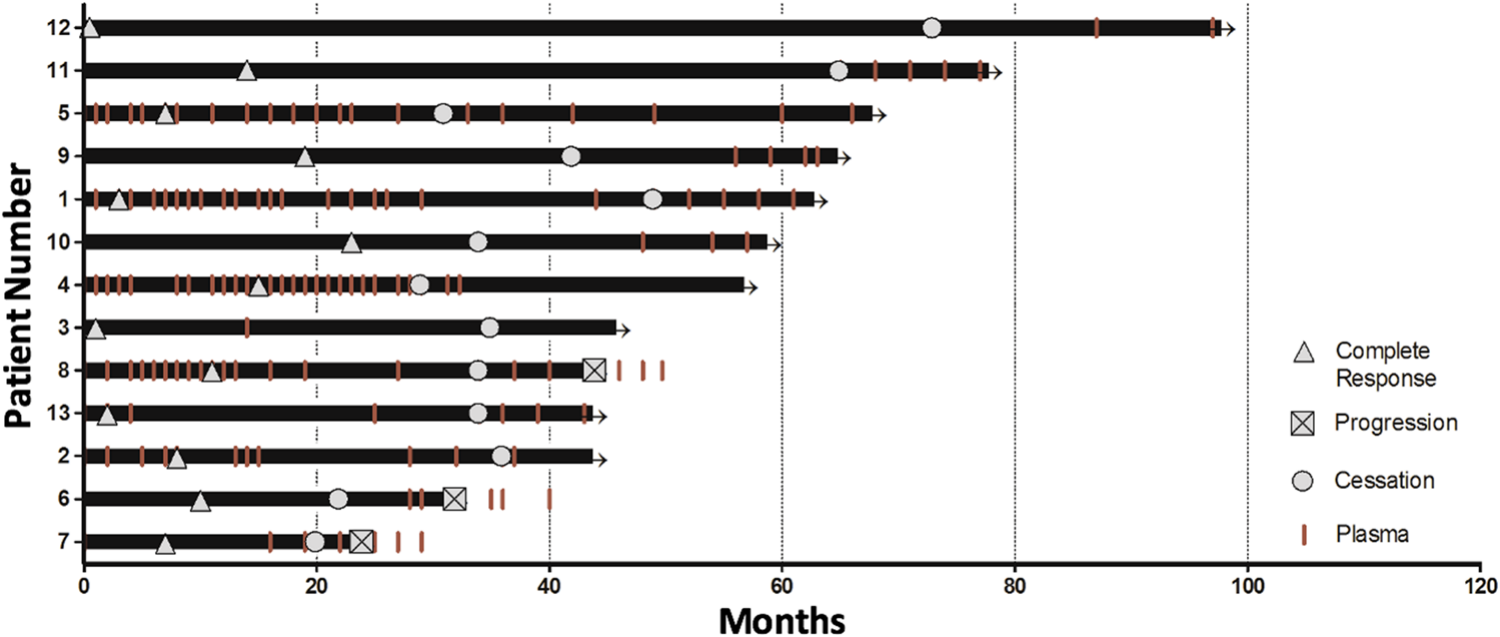
Swimmers plot of all 13 patients treated with BRAF inhibitors. Time on treatment, time to complete response and time off treatment are indicated for each case. Arrows indicate continuation of complete response off therapy. Lines indicate plasma collection time points.

The median observation period, from the commencement of therapy to census date was 57 months and 19 months from cessation of BRAF inhibition (Fig. 1). The average duration of therapy was 39 months (median: 34; range 20–73). The average time on therapy after a CR was achieved was 29 months (median: 24, range 11–73).

### Melanoma recurrence

Recurrence, identified by PET/CT, was observed in three patients (Fig. 1). The median time to recurrence following treatment cessation was 5 months (range 5–11). All three patients had M1c/d melanoma with normal LDH and good performance status at baseline. Recurrence occurred in the encorafenib treated patient and in two dabrafenib and trametinib (CombiDT) treated patients. Two of these patients had recurrence in previous sites of disease, one patient had recurrence in a new site only. Two patients had recurrence in the brain.

### ctDNA analysis

**Figure 2:**
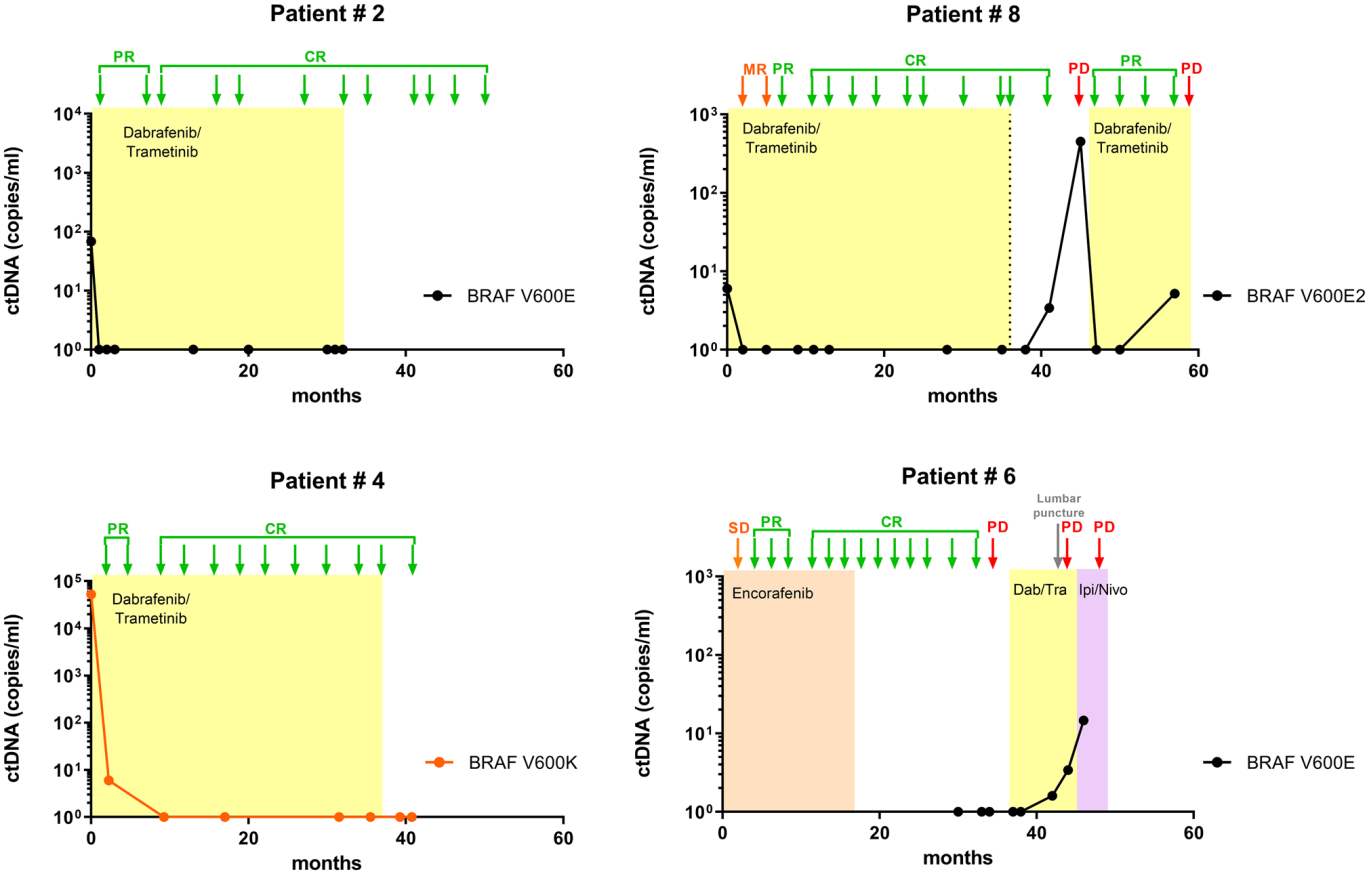
Longitudinal tracking of ctDNA during and post treatment for patients 2, 4, 6 and 8. Time on therapy is highlighted in yellow for dabrafenib and trametinib, orange for encorafenib and pink for subsequent immunotherapy (ipilimumab and nivolumab). Disease assessments by radiological imaging are indicated by arrows and labelled as *SD* stable disease, *PR* partial response, *CR* complete response or *PD* progressive disease.

In total, we analysed 82 plasma samples from thirteen patients for the presence of ctDNA. One patient only provided one sample during treatment. As this was a retrospective analysis, the blood collection time points were not consistent across the cohort. Only four cases had plasma collected at the time of therapy initiation, of those three were positive for ctDNA (Fig. 2). CtDNA levels became undetectable soon after treatment initiation in all three cases, remaining undetectable throughout therapy, at the time of cessation and after cessation.

CtDNA in 36 plasma samples collected during therapy from eight cases had undetectable ctDNA. In addition, we analysed 46 plasmas collected at or after cessation of therapy from 12 of the patients (Fig. 1). Except for three patients who relapsed (detailed below), all samples for patients at the time of treatment cessation and beyond had undetectable ctDNA.

**Figure 3:**
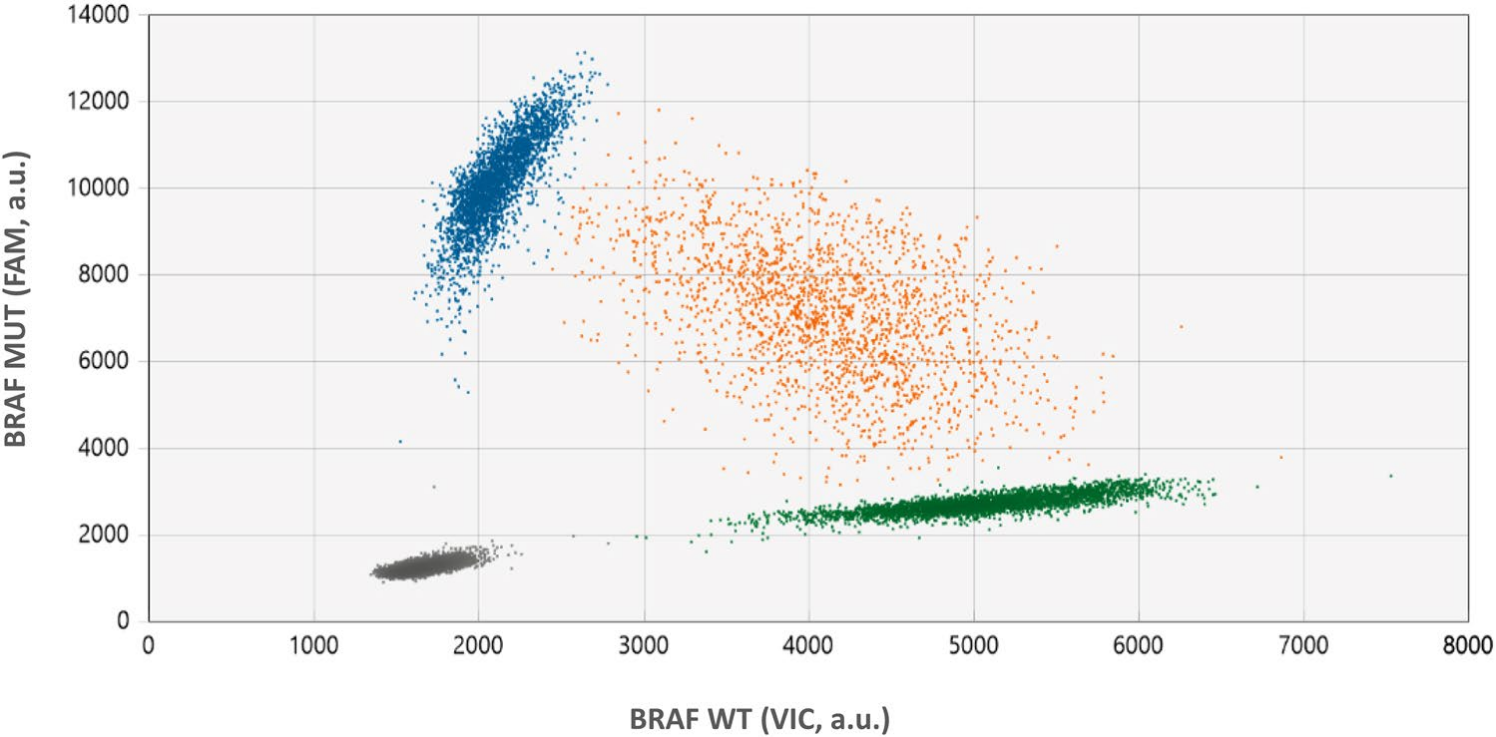
Cerebrospinal fluid-derived ctDNA. Level of ctDNA in CSF in patient #6, who developed leptomeningeal disease after re-challenge with targeted therapy. 2D plot from ddPCR analysis indicates the presence of DNA mutant for BRAF V600E (FAM, blue dots), BRAF wild-type (VIC, green dots) or both (orange dots). *a.u.* denotes the arbitrary unit for FAM and VIC probe intensity.

In patient #6, melanoma recurred 5 months after cessation of encorafenib. He developed recurrence with a single solitary asymptomatic cerebellar metastasis detected on surveillance imaging. He did not previously have intracranial disease. This solitary metastasis was surgically resected and no additional disease was identified on staging scans. CtDNA was not detectable at time of initial recurrence. Unfortunately, samples were not available prior to initiation of therapy to determine if ctDNA was ever measurable at baseline. He was rechallenged with dabrafenib and trametinib achieving PFS of 7 months. He developed leptomeningeal involvement as confirmed by lumbar puncture and MRI brain. It is of note that the ctDNA level detectable in plasma at time of relapse was very low (1.6 copies/mL) but was very high in cerebrospinal fluid CSF (9500 copies/mL) (Figs. 2, 3). He was switched to combination immunotherapy, but unfortunately progressed with leptomeningeal disease with associated increasing plasma ctDNA levels (Fig. 2).

Patient #7 had intracranial and extracranial disease at baseline which responded to combination targeted therapy. Tumour recurrence was detected 5 months after cessation of dabrafenib and trametinib, and occurred intracranially at sites of original disease. No recurrence was observed in the original extracranial organ sites (liver, nodes). CtDNA levels were undetectable at the time of treatment cessation and on relapse. Dabrafenib and trametinib were restarted at full dose without toxicity. Early response assessment demonstrated a good partial response but progression was confirmed on imaging 4 months after rechallenge. She was switched to combination immunotherapy but unfortunately died of progressive intracranial melanoma eight months after relapse.

Patient #8 relapsed eleven months after stopping treatment. At relapse, tumour growth was observed in intraabdominal lymph nodes and was asymptomatic. Dabrafenib and trametinib was restarted and he attained partial response. Ongoing response was observed for 8.5 months after recommencement, with subsequent radiological progression in intraabdominal nodes. CtDNA became detectable 3 months prior to radiological evidence of relapse (Fig. 2).

## Discussion

Here we present a case series of metastatic melanoma patients who stopped BRAF targeted therapy after obtaining CR. Only three out of thirteen patients have relapsed with a median follow up period of 19 months since treatment was stopped. This is the longest published cessation and observation follow up study to date. Although this single centre retrospective study describes a small cohort of patients, it provides further insight into whether BRAF inhibitors can be safely discontinued in complete responders. In addition, it combines a unique perspective by incorporating clinical outcomes with circulating tumour DNA analysis and shows the efficacy of ctDNA as a measure of disease presence.

A 3 year analysis of patients on BRAF/MEK inhibition revealed 58% patients with CR had maintained CR at 3 years; of these, 21% had stopped treatment. No details are available about the median duration of time off treatment^21^. Additionally, at 5 years, progression free survival rate (49%) and overall survival rate (70%) were significantly higher in patients with confirmed CR. At 5 years 23% of patients discontinued the study drug in the absence of disease progression^21^.

Five other publications have described patient outcomes upon cessation of BRAF inhibitors (Table 2). Wyluda et al*.*^22^ described three cases of durable CR without relapse 15 months after treatment was ceased. These three patients were treated for a finite period with BRAF inhibitor after being initially unsuccessfully treated with immunotherapy. The other four publications described experiences with cessation of BRAF inhibitors in their patients. The majority of patients in these four series stopped due to toxicity (54–100%) with a relapse rate of between 46 and 53% after a median follow up of 12–17 months^22–26^.

**Table 2:** Comparison of outcomes of reported cohorts after stopping BRAF inhibitors.

Case series reporting outcomes after discontinuation of BRAFi	Number of patients (CR)	Median duration of BRAFi treatment (months)	Therapy ceased due to toxicity	Median follow up after treatment cessation (months)	Relapse rate (%)	Median time to relapse (months)	Percentage rechallenged that responded (CR/PR)
Wyluda et al.^21^	3	9–12	100%	15	0	N/A	N/A
Tolk^24^	12	13	54%	17	46%	3	50%
Carlino^22^	12	N/A	100%	16	50%	6.6	33%
Vanhaecke^25^	16	21	63%	12	53%	2.5	63%
Desvignes^23^	6	10	100%	15	100%	4	17%
Warburton	13	39	0	19	23%	5	100%

*N/A* not available.

Our cohort differs to those previously published as all 13 patients received BRAF inhibitors as first line treatment and cessation occurred after a lengthy period of treatment in CR, in the absence of treatment limiting toxicity. Our median follow up since cessation is longer and relapse rate lower (Table 2). Vanhaecke et al*.*^26^ described in their cohort that duration of treatment after CR was shorter in relapsing patients. Similarly, three patients in our cohort who relapsed were on treatment for a median of 16 months (range 12–23) after CR vs 29.5 months (11–73) in the non-relapsing patients. Furthermore, the duration of treatment prior to cessation was significantly longer in our cohort compared to others described (median: 39 months) perhaps contributing to the lower relapse rate observed^22–26^. With respect to the 10 patients with ongoing response, it is difficult to appreciate any consistent pattern of clinical characteristics due to the small number. As reported in the literature, good prognostic indicators include good performance status, low number of metastatic sites, no CNS disease and normal LDH^10^. However these clinical biomarkers are prognostic and not predictive and therefore within our small cohort, some patients with brain disease, high LDH and multiple metastatic sites continue to maintain CR despite cessation of therapy.

In the pooled 3-year analysis of clinical outcomes of patients treated with dabrafenib and trametinib 19% of patients achieved CR. Progression after CR occurred in 42%, 39% of whom were not on therapy at time of progression^12^. Further analysis of the subgroup who progressed after CR (n = 106) by Robert et al*.* demonstrated that progression following CR was usually observed in new lesion organs (89%) and most commonly in CNS (54%)^21^. In our relapsing patients, 33% (n = 1) relapsed in a new organ location and 66% (n = 2) relapsed in the brain.

It has been reported that rechallenge with BRAF inhibitors in patients who initially respond is associated with further response and improvement in overall survival. In a series of 116 patients rechallenged with BRAF inhibitors objective response rates were 42% and PFS was 5 months (range 0.2–31.7) which was similar to our patients (median: 7 months, range 4–8.5)^27^. Additionally in Desvigne’s cohort of eleven patients, eight patients were rechallenged with a BRAF inhibitors with a median progression-free survival time of 7.2 months (range 15–425 days)^24^. The first prospective clinical trial demonstrating objective response to dabrafenib and trametinib rechallenge hypothesized that the acquired mutation to BRAF inhibitors could be reversed when the selective pressure of BRAF inhibition was withheld. Patients (N = 25) who were off BRAF targeted therapy for at least 12 weeks were rechallenged after progression. Partial response was achieved in 32% and stable disease in 40%^28^. Our cohort is different as the relapsed patients never experienced evidence of acquired resistance and progression during the primary course of targeted therapy. However, our results confirmed that patients who relapse post treatment can respond to reintroduction of BRAF/MEK inhibitors.

CtDNA is emerging as a promising blood biomarker to indicate initial tumour burden, monitor treatment response, evaluate dynamic mutational changes and predict for minimal residual disease in melanoma and other cancers^18,29,30^. In melanoma, several studies have shown the clinical utility of using ctDNA for tracking response to therapy and prognostication^18,20,29–34^. Baseline ctDNA levels have been shown to be directly associated with radiological tumour burden and inversely associated with response and PFS^34–37^. Low levels of ctDNA are associated with better responses to targeted therapy and reductions in ctDNA levels an independent predictor of response to BRAF inhibitor therapy^18^. Furthermore, Lee et al*.*^30^ and Cabel et al*.*^38^ have both shown that undetectable ctDNA at baseline or within eight weeks of commencing anti PD1 therapy is an independent predictor of response and PFS. There have been no studies to date that investigate ctDNA as an indicator or predictor for safe early cessation of therapy and durable PFS.

Lee et al.^39^ published compelling data demonstrating that ctDNA can detect minimal residual disease which also corresponded to poorer outcome and survival in patients with high risk stage II and III resected melanoma. Similarly, in breast and colon cancer, as well as more recent melanoma studies have again shown that detectable ctDNA after curative resection is associated with recurrence and worse survival^15–17,40^. Analysis of ctDNA in patients who are stopping BRAF-inhibitors may reveal which patients have minimal residual disease and would benefit from ongoing targeted treatment. All 13 patients who contributed blood samples had undetectable ctDNA either during, at completion or after cessation of therapy. Unfortunately it appears that a limitation of ctDNA is in its detection of intracranial disease, likely accounting for the unmeasurable levels in both patients #6 and #7 who recurred intracranially^41–43^.

There appears to be a subgroup of patients, as demonstrated in our and previously published series, that attain CR and maintain it after treatment is stopped. Our retrospective study has a number of inherent limitations including the small sample size, the inconsistent blood collection time points across the patients and the absence of blood collection and analysis prior to commencement of treatment for eight of the patients. Clearly with no prospective data available to guide treatment cessation decisions, stopping BRAF inhibitors in CR cannot be widely recommended. Our study gives credence to the fact that further studies about treatment cessation are required in patients who have good prognostic clinical features, have tolerated a prolonged treatment course with CR (> 2 years) and have undetectable ctDNA. Currently there are no clinical or biomarker correlates that assist clinicians in counselling patients to cease or continue treatment. Our study shows promise that ctDNA may aid these decisions. However, ctDNA has inherent limitations in patients with low volume disease and brain metastases where it can remain undetectable despite relapse. Larger, prospective studies are needed to select appropriate patients and optimise the timing of treatment discontinuation, and the incorporation of novel surrogate endpoints such as ctDNA in clinical decision-making, with the ultimate aim to mitigate unnecessary toxicity and treatment expense.

## Data Availability

All data generated or analyzed during this study are included in this published article and its supplementary information files.
